# The Provident Dispensary

**Published:** 1899-03-04

**Authors:** 


### THE PROVIDENT DISPENSARY.

We all wish well to the provident dispensary system, although we have at the same time to admit that so far it has not proved an unqualified success. The difficulties in the way of the system are two-fold; the poor, for whose benefit it is meant, often decline to be provident, at any rate so far as medical attendance is concerned, while the well-to-do, for whom it is not meant at all, jump at it as a cheap and easy way of having a doctor always at their beck and call, and unfortunately they are frequently accepted as members. These points are well brought out in an article on "The Dispensary" in this month's *Charity Organisation Review*. The British workman, it says, pays a penny into the Hospital Fund and considers he has done his duty. As for the provident medical system, he finds nothing tangible at the end of it. If it were a goose or a slate club, there would be at the end something to eat or drink. If it is a burial club, then the knowledge that if the baby does die it will have an extravagant funeral: a hearse with white plumes, and something over to spend on crape, and a feast of baked meats. But the Medical Provident—if we are ill we can have a doctor. "Can we? Well, the 'orspital doctor is good enough for us." The fact is that, "what with free medicine, free dinners, free boots, country holiday funds, and the like," thrift cannot be expected. "Some years ago I was attending from the dispensary a child with inflammation of the lungs. One day, when visiting the house, I found the husband sitting half drunk by the fire. I asked him if he was out of work, or on strike. 'Oh, no,' he said; 'but I won ten pounds on Ragimunde for the Cesarewitch, and I'm having a day off.' It never occurred to him to offer to pay a doctor, not a bit." Speaking of the intrusion of the well-to-do into the provident system, the writer says: "The Metropolitan Provident Medical Association have done a great work, and I am proud to be one of their officers; but I consider they have discounted their original principles by affiliating under their rules certain old-established clubs without inquiring into the circumstances of the members of those clubs." From the medical side this is the real difficulty. It seems impossible to persuade secretaries and committees that when, for the sake of enlisting steady subscribers, they admit those of a higher class they are really cheating the profession. But such things need not be if the profession would establish provident clubs or dispensaries under purely professional management.

